# Using the Delphi Approach to Identify Priority Areas for Health Visiting Practice in an Area of Deprivation

**DOI:** 10.1155/2013/780315

**Published:** 2013-09-09

**Authors:** Rosamund Bryar, Sandra Anto-Awuakye, Janice Christie, Claire Davis, Karen Plumb

**Affiliations:** ^1^School of Health Sciences, City University London, Northampton Square, London EC1V 0HB, UK; ^2^Community Health Services, Barts Health NHS Trust, 9 Prescot Street, London E1 8BR, UK

## Abstract

Families with children living in areas of high deprivation face multiple health and social challenges, and this high level of need has impacts on the work of health practitioners working in such areas. All families in the UK with children under five years have access to health visiting services, and health visitors have a key role in mitigating the effects of deprivation by addressing health needs through evidence based practice. This paper reports the first stage of a project in Tower Hamlets, London, an area of significant deprivation, which aims to develop an evidence-based toolkit to support health visitors in their practice with families. The first stage used a modified Delphi process to identify the priority health needs of families in the area between June and July 2012. The three-stage Delphi process involved 25 people: four health visitors, four other members of the health visiting service, and 17 representatives of other services working with families. A focus group event was followed by a second event where individuals completed a questionnaire ranking the 27 priorities identified in the first event. The consultation process concluded with participants completing a second questionnaire, by email, confirming or changing their prioritisation of the topics.

## 1. Introduction

Development of methods to enable practitioners to use evidence in practice has had increasing focus within the evidence-based health care movement and in all areas of the NHS, with the aim of delivering high-quality care [1]. Prior to the development of interventions in an area of practice, there needs to be an understanding of the priorities for practice in that particular field [2]. This paper reports on the first stage of a six-stage project which aims to develop a toolkit of resources for evidence-based practice in health visiting, in an inner city area, characterised by high levels of deprivation and a high under 25s population. The first stage involved identification of the priority areas of practice for health visitors (HVs) working with families with children under the age of five years.

The project is being undertaken in Tower Hamlets, an inner city borough in east London, UK. Tower Hamlets is a densely populated area of eight square miles characterised by multiple aspects of deprivation which impact the health and wellbeing of the population. The borough is divided into 17 administrative wards and 16 of these are in 20% pf most deprived wards in England and 12 are in the lowest 5% [3]. Of the approximate 250,000 population, 50,000 are under the age of 16 years and of these 25,000 are living in poverty: this is the highest level of child poverty in England [4]. The population is highly mobile and contains a large number of migrants with a population made up of 50% White, 36% Bangladeshi, 7% Black, 3% Chinese, 2% Indian, and 4% other. The joint strategic needs assessment identifies that the effects of deprivation are evident in children in the area who are under the age of five years. To address these effects, it is recommended that the local Health and Wellbeing Board needs to consider that “good education, access to childcare, and support to families are evidence-based interventions to give Tower Hamlets infants the best start in life and mitigate these impacts” (page 21) [3].

## 2. Health Visiting

Health visitors are qualified nurses or midwives who undertake an additional qualification at either BSc or MSc levels to register with the NMC as specialist community public health nurses (HV). The roots of health visiting lie in the concern in the 19th century to improve the health and living conditions of families living in poverty in the UK [5, 6]. HVs are part of the front line public health workforce, visiting every family who has a new baby, focusing on prevention and promotion of health [7]. Through this focus, HVs contribute to reducing long-term health inequalities and prevention of long-term conditions as well as supporting parents with their immediate parenting concerns and working to identify and prevent child safeguarding issues. 

The role of HV services was clearly outlined in the *Healthy Child Programme* [7] and *Healthy Lives, brighter futures*—*the strategy for children and young people's health* [8]. These documents emphasise intervention during the early years of a child's life, identification, and management of risk, as well as universal service provision (care provided to all families with young children) with additional targeted and specialist service provision offered to families in most need. Recent high profile reports [9–15] highlight a critical role for HVs in delivering evidence-based early, preventative intervention programmes for children, young people and families, particularly for those at risk or with identified health and social needs. 

Health visitors are pivotal in improving public health outcomes for children and families [8, 16, 17]. They work in partnership with parents and voluntary and statutory agencies and use social models of assessment. Supporting families in complex situations they identify their strengths and resilience working with them to deliver interventions which promote health and wellbeing, tackle inequalities, prevent illness, and improve social inclusion [18]. High rates of child poverty and social disadvantage greatly increase health practitioners' workloads [19], but it has been suggested that traditionally health visiting services were not optimally planned and offered to tackle social disadvantage [20]. Thus, there is a need for more effective use of HVs' skills to support population needs arising from social disadvantage. 

Due to a combination of factors, the numbers of HVs in practice in the UK dropped significantly from the 1990s. During this time, there was a considerable increase in the evidence concerning the importance of the early years of a child's life on their intellectual and social development [9, 10]. Since the election of the UK coalition government in 2010, there has been a renewed interest in the role of the HV in supporting parents to enable the best outcomes for their young children. In 2011 the *HV Implementation Plan*. *A Call to Action* [17] was launched in England setting out a new model of service delivery and a plan to increase the numbers of HVs by 4,200 by March 2015. The HV Implementation Plan [17] outlines four levels of service delivery by health visitors: universal, universal plus, universal partnership plus, and the community levels, and the Department of Health commissioned the National Nursing Research Unit, King's College London, to examine the evidence demonstrating health visiting effectiveness within these service levels [21]. The report provides a review of the evidence relating to areas of health visiting practice including support for breastfeeding, support for parents and parenting, work with families experiencing domestic violence, and multiagency working. The review shows that in some areas of practice, for example, the role of the health visitor in supporting continuation of breast feeding, there is strong, robust research evidence of effectiveness of health visitors, but in many other areas the evidence base is weak; that is, there is a lack of research in many of the areas of health visiting practice. However, in relation to the early intervention work that health visitors are undertaking with families, there is an extensive evidence base including the recent reports from the WAVE Trust [22, 23] which summarise the evidence on the importance of early intervention for the long-term wellbeing and health of infants, children, and families. The question underpinning the present study was: to what extent are health visitors using the currently available evidence in their work with families?

To support the expansion in the numbers of health visitors The Burdett Trust for Nursing launched a funding round to promote innovative developments to empower HVs in practice. Funding was granted by The Burdett Trust for Nursing to develop a toolkit to support HV practice in an inner city area. HVs who work in deprived inner city areas (such as Tower Hamlets, East London), with high levels of mobility, vulnerability, and safeguarding concerns, are constantly making difficult decisions about how they prioritise their workloads. They are faced with limited workload capacity due to large and growing under 5 population and caseloads which have disproportionate numbers of vulnerable families, or families with child protection concerns. As a result, it is hard for HVs to focus on the core universal health visiting role which aims to improve the health of the whole population by intervening early in the lives of children and families to prevent ill health or deterioration. 

## 3. Evidence-Based Practice

As Nursing and Midwifery Council registered nurses, HVs are required to ensure that they provide safe, effective, and evidence-based care [24]. Evidence-based practice (EBP) is a means of ensuring that effective and safe interventions are delivered [25], but EBP does not just refer to using research in practice [26] since research is only one form of evidence used by nurses [27]. Nurses also use information from their local environment, patient, and clinical experience to inform their evidence-based decision making [28]. 

Currently there is no universal agreement about how research for EBP is used, applied, or evaluated in practice [29]; however, it has been suggested that managerial support, colleague support and education can encourage the use of EBP [30, 31]. Nurses, working in contexts where effective practice is promoted by leadership and support, report significantly more research utilisation, staff development and lower rates of patient adverse events than nurses working in less positive contexts [32]. It is suggested, therefore, that leadership, clinical support, and reflective practice make EBP “thrive” [33]. Eraut [2] provides a framework in which the utilisation of EBP may be considered. He describes three dimensions which interact to inform practice as follows:

- the first is concerned with the analysis of needs in practice including assessment, planning, and implementation;
- the second is concerned with the context of practice, for example, cultural aspects, deprivation;
- the third is concerned with how the professionals think which is affected by their experience and the time available.

The present project is concerned with examination and intervention in each of these areas with the aim of enabling HVs in Tower Hamlets to incorporate EBP into their work with families with significant need. HVs have limited time to access research evidence, and therefore the project aims to develop a resource or toolkit which would provide the HVs with easily accessible resources to support their practice. Research on barriers to research utilisation indicates that lack of time is the key barrier to use of research by practitioners [30], and, where toolkits of resources have been put in place improved patient outcomes have been achieved [34]. However, the content of the “toolkit” has evolved over the project reflecting the reality of introducing change in practice [35] so that the final toolkit will, in addition to evidence for practice, contain other resources supporting, for example, communication, multiagency working, and teamwork.

Development of the toolkit has been led by a six-cycle process of research making use of an action research approach [36] as follows:

- 1st cycle is identification of priority needs and development of consensus among stakeholders about the top priority health needs to be addressed through the EARLY toolkit by HVs in Tower Hamlets.
- 2nd cycle is identification and synthesis of evidence-based material that will provide best evidence to support service delivery regarding the top priority health needs.
- 3rd cycle is identification of past and current HV activity regarding the top priority issues.
- 4th cycle is development of the EARLY toolkit for the priority areas and HV training in use of the tools.
- 5th cycle is an evaluation of changes in practice (in comparison with data collected in cycle 3) after implementation of the EARLY toolkit.
- 6th cycle is embedding of EARLY toolkit outcomes into information systems in order to support future audit of activity for the top priority issues.

The project was submitted to the Research Office of Barts Health NHS Trust and on the advice of the Trust, it was registered with the Clinical Effectiveness Unit in the Trust as a service development project. The project is overseen by a steering group chaired by the Head of Nursing Community Health Services (KP) which meets every two months. The steering group includes representatives from public health, the safeguarding children's team, health visiting in the two pilot localities in which the work is being undertaken, the children's centres lead and the project manager (CD), and the university research team (RB, SA-A, JC). The steering group reports to the project sponsor in the Trust. An operational group led by the project manager and the university researchers meets at least every fortnight.

## 4. Analysis of Needs in Practice

The overall aim of the project is to develop an EBP toolkit that will inform practice and benchmark safe and effective early years' HV interventions, thereby supporting service development and audit. It was proposed that the toolkit would contain resources to support delivery of EBP by HVs in priority areas. The first step (cycle 1) was to identify the most important issues or top priority needs that the HV team should address, to prioritise the development of the resources.

Various methods may be used to identify priorities in practice including reviewing the literature, examining the local joint strategic needs assessment and public health data, and consultation methods. As the long-term aim of this project was to develop resources for HVs to use in their practice, it was important to involve HVs and other service providers who they work with in the identification of priorities. To do this, the project made use of a modified Delphi process to identify priority issues in HV practice in an inner city area.

## 5. Modified Delphi

Delphi is a structured process that uses a series of repeated rounds to gather information from a panel of experts. The aim of using Delphi is “to achieve agreement among a group of experts on a certain issue where none previously existed.” [37, page 4]. Each round summarises information presented in the previous round which is then presented again to stakeholders for prioritisation in order to establish group agreement [38]. Delphi is usually undertaken in 3 rounds conducted by post and agreement among panel members is achieved by providing each member with feedback and averaged information from the previous round [39, 40]. 

The technique was developed in the 1950s as a means to facilitate the engagement of experts as a group to examine complex defence problems in the USA [41]. These authors provide a general definition but stress, as do others, that Delphi is an approach rather than a fixed method: “Delphi may be characterised as a method for structuring a group communication process so that the process is effective in allowing a group of individuals, as a whole to deal with a complex problem” [41, page 3]. Delphi may be used for two main purposes: priority setting or gaining consensus on an issue.

Keeney et al. [37] identify 11 different types of Delphi which have emerged over time from the original or classical Delphi. The modified Delphi they describe has a first round involving the expert group in face-to-face interviews or focus groups. In the case of the present study, the aim was to identify priorities to be addressed through the EBP toolkit. A modified Delphi approach was used in which the first round comprised focus groups, the second individual responses but in a group setting and the third was conducted via email.

## 6. Method

The aim of the first cycle was to identify, explore, prioritise, and gain stakeholders' consensus about the priority health needs that HVs will address through the EBP toolkit, making use of a three-round Delphi approach.

## 7. Participants

Purposeful samples of people from health visiting and the stakeholder groups who work with the same population of families with children under 5 years were invited by KP to participate in the three rounds. Information was sent to potential participants outlining the aims of the project and detailing what was required from them. Health visitors in the two localities, of four, in which the project was to be piloted were invited to attend. The two localities reflect the widespread nature of deprivation in the borough and the ethnic mix of the population. The participants were service representatives from across the borough; their invitation and involvement in the process were to ensure that participants were people who had the commitment to the process and shared a level of expertise. For example, participants were diverse, ranging from an HV team member to manager in a children's centre (see Table 1). The project was initially planned to run for one year which meant that a very tight timetable for the Delphi process was followed resulting in a short period for recruitment of participants for the Delphi process from the start of the project in mid-May 2012 to the focus group in early June. This resulted in recruitment of only one parent but 21 parents have been involved in later stages of the project. Participants were asked to agree to participate in all three rounds of the Delphi, and at the beginning of Round 1 they were asked to sign participation consent form.

### 7.1. Round 1

The participants were invited to attend a one-day event in June 2012 held in a Trust venue during which the aim was to generate discussion and collect qualitative data about the priority areas. Refreshments and lunch were provided during the day. The day started with a presentation on the project, and then participants were formed into five focus groups each led by a facilitator. Each focus group had 4–6 participants, an appropriate sample size for a focus group [42]. The focus groups included people from the same professional groups or services; for example, children centre and outreach workers remained in one group, and allied health professional teams (stakeholders) and health visiting teams had their own groups. The parent who attended joined the group of children centre and outreach workers as she agreed this group was most acceptable to her. The role of the facilitators was to develop and generate group discussion surrounding their work with health visiting services and families with young children under five years. Participants were also asked about their perception of health visiting services, how they work with the services, and their thoughts on what are the greatest needs seen in families and children in the services they provide (for nonhealth visiting groups). Each facilitator was provided with a guidance document appropriate to the particular group they were facilitating (see Table 2 for one example of the guidance to facilitators). 

The discussions in the focus groups were taped and notes made on flip charts summarising the discussions, and prioritising the identified needs. The key points from the discussions were fed back verbally to all the participants at the end of each discussion period through the day.

The tapes were transcribed and the flip chart notes were typed up by the research team following the event. Content analysis was used to identify the topics which the participants had identified as the priority areas of health visiting practice to be addressed through the toolkit [43].

### 7.2. Round 2

A questionnaire was developed listing each of the priority topics with a range of comments illustrating the rationale for the topic as described by the participants. The participants from Round 1 were invited to attend a second half day in June 2012, two weeks after the first session. Following a brief update on the project, each participant was asked to read each topic and comments from Round 1 and to rank each topic as a priority from 0 not important to 10 very important. The numerical rating system aimed to test the extent to which the group agreed or formed a consensus around the most important areas to be addressed in the toolkit. Participants were also given the opportunity to add a brief rationale for their decisions if they wished. The topics and individual participants' scores were entered onto a spread sheet, and mean scores for each topic were calculated. This generated an initial list of prioritised topics.

### 7.3. Round 3

A questionnaire, individualised for each participant, was constructed covering all the priority topics and sent to participants via email in July 2012. In this questionnaire, the participants' individual score for each priority area was shown alongside the mean score from the group for that topic. Comments explaining the rationale for the topic, from the individual participant and other participants, were also included. The participants were asked to confirm their previous score for the topic or to change it in light of the mean score and the comments. In the third round, the participants were thus given the opportunity to reprioritise the priority topics.

## 8. Results and Discussion

The results from the three rounds are presented below in the order in which they occurred. The majority of the 25 participants took part in the three stages of this study, with 23 responses from 25 participants in Rounds 2 and 3. One participant was not present at Round 2 but undertook the online questionnaire at Round 3. Another participant completed the Round 2 questionnaire but not the one in Round 3.

### 8.1. Round 1

Twenty-five people attended the Delphi event representing health visiting teams and a wide range of stakeholders. Group discussion took place in several sessions over the day, each lasting for up to 2 hours, with the objective that at the end of the day each member would feel that their own priorities were properly represented on the list of priorities. Discussions were vibrant, interactive, and revealing and covering a wide range of issues from how health visiting services are perceived to how allied health professionals and children centres endeavour to work with families more closely. One participant from Children and Adolescent Mental Health Services (CAMHS) stated, *“We feel underused… [health visitors should] feel free to refer babies as young as 2 months to CAMHs but nobody knows”*. This discussion provided an opportunity to learn about how another service like CAMHS complements the work being achieved in health visiting such as early intervention strategies for maternal mental health. In the children's centre group, there was a perception of HVs being constantly busy and it was observed that *“lots of HVs are coming and going”* leading to a lack of consistency for clients. These issues were raised as potential barriers to building sustainable relationships between health visiting and children's centre staff; however, both groups recognised the value of working together to achieve positive outcomes for families.

Each group identified major social issues such as poor housing, unemployment, social isolation, and difficult family circumstances linked to fragmented family relationships, difficult marriages, and problems with extended family as impacting the work of HVs. These concerns were recurrent themes in daily work with families; participants asked if an issue such as housing was worth identifying on their priority list as HVs have limited capacity to influence these kinds of social issues. It was agreed that all issues however great or small would be identified by the groups to ensure a true representation of their views about the priorities.

The discussions generated a large volume of data and some of which is illustrated in Table 3.

Round 1 led to the generation of 27 priority topics (see Table 4). The purpose of Rounds 2 and 3 was to generate the priority ordering of the health needs.

### 8.2. Round 2

The questionnaire completed at the second meeting by individuals asked participants to give a priority score for each of the 27 topic areas from 1 to 10. The following provides an example of a questionnaire entry, on the topic of play, including representative statements from the discussions in Round 1 illustrating the topic.

*Example Section from the Round 2 Questionnaire Completed by All Participants*

*Play*. We need some form of play sessions which could be done in conjunction with children's centre.

We wish that we could do more play activities as most people live in high-rise flats and the children are living indoors.

Children's behaviour is really poor in hospital; they are stuck in a flat with lack of stimulation. When the toy library went, that created a real gap.                 Unimportant 0                1                2                3                4                5                6                7                8                9                Very important 10


Please comment on the reasons for your answer

 ……………………………………………

The numerical rating system aimed to test the extent to which the group agreed or formed a consensus around the most important areas to be addressed in the project. A list of the 27 topics in priority order was generated by entering the results from the questionnaires into a spread sheet. A mean score for each topic was generated from the 23 responses in Round 2 (the same procedure was followed in Round 3) giving the list of topics in priority order.

 Participants were also asked to add any additional comments to justify their scores.  Table 5 illustrates some of these comments in relation to three topics.

### 8.3. Round 3

Individualised questionnaires for each of the 25 participants were constructed for Round 3. These included additional comments made by the group and the individual during Round 2.

*Example of a Round 3 Questionnaire Emailed to Participants*

*First-Time Mothers*. First-time mothers need more support. Round 1 Focus Group. 

             Your personal score from 27th June  9

             Mean score of the group       7.6

*Some General Comments Made from the Group, June 27 2012 [Round 2 Questionnaire].* Depends on support networks around them. Some first time mothers will have support of family, friends and neighbours. Others who are more isolated may need more access to advice/information and support.

I see a lot of first-time mothers who are very socially isolated and do not have family support. They present with problems frequently. This is a problem that crosses both cultures and social class boundaries.

Each individual needs to “be” assessed in relation to support networks, understanding and expectations of parenting, and so forth.

*Your Personal Comment*. I see a lot of first-time mothers who are very socially isolated and do not have family support. They present with problems frequently. This is a problem that crosses both cultures and social class boundaries.

*First-Time Mothers: Today's Score*.               Unimportant 0               1               2               3               4               5               6               7               8               9               Very important 10


Participant 12.

The response rate was high with 23 of the 25 questionnaires returned. There was little change in the mean scores across the 27 topics suggesting a strong consensus among the stakeholders about the priorities. The order of the prioritised health needs remained the same as that in Round 2. The priority order is shown in Table 4.

The topics identified through the Delphi process cover a wide spectrum of health and social needs indicative of the level of deprivation in the area and pressures on families with children. The steering group discussed the list of topics and came to the conclusion that it would be impractical to address all of them at the same time given the time scale of the project (at that stage one year later extended to two years) and the resources available. Consideration of the list of ranked topics showed that a number were closely associated, for example, topics 1 and 3, infant stimulation and speech and language. Discussion in the steering group also identified topics which were the focus of local initiatives and development, for example, topic 2 domestic violence, and it was agreed that this work should be incorporated into the toolkit in due course. It was agreed that a number of the topics could be amalgamated into three priority areas to be addressed in the next stages of the toolkit development:

1. infant stimulation and speech and language (topics 1 and 3),
2. preventing obesity (topics 6, 7, 14, and 16),
3. stressed and unsupported families (topics 4, 5, 12, 13, and 18).

## 9. Conclusion

The use of the modified Delphi technique allowed a participative and inclusive approach that encouraged all the stakeholders to influence the selection of priority needs. It promoted consideration of the three elements of EBP utilisation outlined by Eraut [2] and led to identification of 27 priority topics from a variety of stakeholders' perspectives that included consideration of their context of practice and experiences of service delivery. The process was also successful in engaging people through the three stages of the consultation process as 23 participants completed all three stages. 

The prioritised list of topics is the list identified and then ranked by a group of HVs and other practitioners working with families in an area of high deprivation in east London. The highest ranked topic, infant stimulation and speech and language reflects the local concerns but also national policy concerned with the importance of the early foundation years [7, 12, 22]. A quarter of year 6 children in Tower Hamlets are classified as obese, above the average for England [44], and therefore the ranking of prevention of obesity as the second highest priority reflects local needs. There are many challenges to families in the borough including high levels of poverty, unemployment, deprivation, and environmental challenges including congested housing and high traffic flows [3, 44]. Many national reports have identified the pressures on families facing such challenges and thus the identification of stressed and unsupported families as the third priority reflects the high need in the area but is also in accordance with national findings and policy [9, 10, 12]. This list provides clear guidance for the next stages of development of the toolkit for health visitors in this area. It would be interesting to explore in other areas, with similar or different levels of deprivation, if the same or a different list of priority topics would be generated.

The next stages of the project involve examining the literature for evidence of best practice in the three amalgamated topic areas, collection of data on the use of this evidence in practice through observation of HV-client interaction, interviews with parents and HVs, and examination of electronic records. This will be followed by development and implementation of the EARLY toolkit and evaluation of its use in supporting HVs in their practice with families with children under five years.

## Figures and Tables

**Table 1:** Delphi participants representing stakeholder groups.

Professional groups	Numbers
HVs	4
Nursery nurses	2
HV Managers	2
Children centre workers	4
Children centre managers	1
Breastfeeding coordinator	1
Speech and language therapists	1
Children's dietician	1
Children's physiotherapist	1
GP	1
CAMHS psychotherapist	1
DV coordinator	1
Tower Hamlets public health	1
Local parent	1
NHS Trust managers	2
Health advocate NHS	1

Total	25

**Table 2:** Facilitation guidance for stakeholder focus group. Introduction: we have some questions that we have prepared to help you think through what people in Tower Hamlets need and what services HVs should offer. However, we don't want these to limit you in anyway. Please feel free to discuss things that you think are relevant. Note to facilitators: key questions are in the left-hand column and should be written on your flip charts in advance of the discussion.

Key questions	Triggers if required
What types of needs to you see or know about in your area? What gaps do you notice in current health and social service provision for families with young children? What gaps do you notice in public health provision for the local community?	Tell us about local health and social needs in Tower Hamlets. Tell us about the things that you think impact family and community health for example, practical/social issues: housing, legal advice, schools and education, lifestyle issues, physical health problems, and emotional and therapeutic support. What families have the greatest needs? And why? What communities have the greatest needs? And why? What services are offered and are they well received? And why? What services are poorly resourced? What could be done about these?

What are HVs/health visiting teams currently doing in your area?	How does your service interface with health visiting? What things do families report that HVs do for them? What things do HVs do well? What things could be improved? Is there a difference between your personal experience of HV compared to your professional experience? What are your experiences of working with skill mix teams? Nursery nurses, registered nurses, HV assistants

Tell us about what HVs/HV teams could do to support local community and families?	What types of needs could HVs/health visiting teams meet? How could they meet these? Advice? Clinics? Assessment? Therapeutic interventions?

In your opinion what are the most important issues or top priority needs that HVs/health visiting team should address?	What are the most important things for HVs to do?

**Table 3:** Examples of focus group statements related to the priority health needs.

Topic	Example statements from Round 1
Lack of iron and vitamins	I facilitate weaning groups, even those who are fluent they do not know about the issue of vitamin D deficiency. People are not aware that there is not enough light in the UK, and they do not know that they need to give vitamin drops until age of 5. And the mothers are not aware they themselves are also vitamin deficient, and sometimes the prescription they get from the GP contains gelatin and they cannot use that and the GPs often are not aware of that. And when they are pregnant, there is a risk to the child as well. So that is it; in Tower Hamlets there is a big issue about vitamin deficiency. (Health visitor)

Mental health	One of the main issues is lower mood and depression amongst men and women. I am seeing it more and more in men. I do not understand why, could be financial, but I am seeing a lot of that and we are doing a lot of work around that. We are doing work with mothers to start off with, who are not clinically depressed for people who feel like sleeping all the time, and refuse to have an assessment done because of the stigma of being diagnosed with something. (Outreach children centre manager)

Speech and language	I have worked in TH for 7 years, I'm a child psychotherapist from CAMHSs. One of the needs that really sticks out in my mind is to support parents need at the very beginning of a child's life. This is concept that you do not need to talk to babies. In CAMPHs, we come across mothers who say, “I did not know you talk with a baby”. Now the baby is 2 years old with no ability to speak or interact and being mistaken as autistic features. Encourage Mothers to interact, even best meaning mothers say, “I did not know you talk with a child, play with a child, look into the child's eyes”. So not surprisingly, they do not know how to interact mothers interacting even in a basic way to play. Unfortunately, it is inability for parents to interact. I think that collaboration between CAMHS and health visitors is crucial. (CAMHS: clinical psychologist)

**Table 4:** Priority needs for Health Visiting as identified by participants in Rounds 2 and 3 of the Delphi process.

No.	Topics
1	Infant stimulation
2	Domestic violence
3	Speech and language
4	Vulnerable children and families
5	Mental health
6	Overfeeding/force feeding/obesity*
7	Breastfeeding/infant nutrition
8	Families with no recourse to public funds
9	Behaviour
10	Poor uptake of services
11	Physical development
12	Social support, isolation, and emotional wellbeing
13	Parent relationships
14	Weaning
15	Housing
16	Healthy eating
17	Play
18	Unemployment and socioeconomic deprivation
19	Lack of iron and vitamins
20	Dental caries
21	Within population needs
22	Addictions
23	Health promotion for families
24	First time mothers
25	Sexual health
26	Children with disability/additional needs
27	Schools**

*Included as one category as statements concerning all three areas from participants indicated a relationship between overfeeding/force feeding and obesity.

**This category referred to the lack of school places in the borough and the difficulties this presented for parents with several children who had to make arrangements to transport children to different schools in different areas.

**Table 5:** Additional comments returned in the Round 2 questionnaires.

Topic	Example statements from Round 2
Behaviour	By educating parents on strategies to managing behaviour which can be applied to all areas for example, feeding/toilet training/child development, and so forth. Then this will surely result in preventing a number of problems we see with the parent/child relationship. (HV1)

Housing	Huge problem in borough having impact on development, constant moving, ability to parent, disrupted networks (professional and social), and health. We are not able to change the family's housing situation, however, we can offer advice on how to manage in the circumstances they are living in for example, signposting to services, educating on importance of play, and so forth. (HV2)

Infant stimulation	Bonding/attachment is critical at an early age. Secure attachment is part of the foundation of making relationships. The basis to emotional, social, and physical development and attachment which needs to be established in the early years. (HV1)
